# Sedentary behaviors and anxiety among children, adolescents and adults: a systematic review and meta-analysis

**DOI:** 10.1186/s12889-019-6715-3

**Published:** 2019-04-30

**Authors:** Bartlomiej Stanczykiewicz, Anna Banik, Nina Knoll, Jan Keller, Diana Hilda Hohl, Joanna Rosińczuk, Aleksandra Luszczynska

**Affiliations:** 1 Department of Nervous System Diseases, Wroclaw Medical University, Bartla 5 Street, 51-618 Wroclaw, Poland; 2 Wroclaw Faculty of Psychology, SWPS University of Social Sciences and Humanities, Ostrowskiego 30b Street, 53-238 Wroclaw, Poland; 3 Department of Education and Psychology, Freie Universität Berlin, Habelschwerdter Allee 45, 14195 Berlin, Germany; 4 Trauma, Health, & Hazards Center, University of Colorado, 1420 Austin Bluffs Pkwy, Colorado Springs, CO 80918 USA

**Keywords:** Anxiety, Sedentary behaviors, Children, Adolescents, Adults, Systematic review, Meta-analysis

## Abstract

**Background:**

Although the number of studies examining the relationships between sedentary behaviors (SB) and anxiety is growing, an overarching evidence, taking into account children, adolescents, and adults as well as different types of SB and different categories of anxiety outcomes, is still missing. Thus, this systematic review and meta-analysis aimed at obtaining a comprehensive overview of existing evidence.

**Methods:**

A search in the following databases: PsycINFO, PsycARTICLES, Academic Search Complete, ERIC, HealthSource: Nursing/Academic Edition and MEDLINE, resulted in *k* = 31 original studies included in the systematic review (total *N* = 99,192) and *k =* 17 (total *N* = 27,443) included in the meta-analysis. Main inclusion criteria referred to testing the SB--anxiety relationship, the quality score (above the threshold of 65%), and the language of publications (English). The study was following the PRISMA statement and was registered at PROSPERO (CRD42017068517).

**Results:**

Both the systematic review and meta-analysis indicated that overall average effects were small: higher levels of symptoms of anxiety were associated with higher levels of SB (weighted *r* = .093, 95% CI [.055, .130], *p* < .001). Moderator analyses indicated that trends for stronger effects were observed among adults, compared to children/adolescents (*p* = .085).

**Conclusions:**

Further longitudinal studies are necessary to elucidate the predictive direction of the anxiety—SB relationship and to clarify whether the effects depend on the type of anxiety indicators.

**Electronic supplementary material:**

The online version of this article (10.1186/s12889-019-6715-3) contains supplementary material, which is available to authorized users.

## Background

Sedentary behavior (SB) is reflecting the low end of physical activity and may be placed between sleep and light activity on the movement and energy expenditure continuum [1]. SB involves low levels of energy expenditure (1.0 to 1.5 of metabolic equivalent of task [MET]), usually occurring while sitting, during work or leisure activities, including screen behaviors (e.g., TV watching), hobbies (e.g., reading books), lying down, in transit, or during driving a car [1–3]. SB may be operationalized as the total sitting time per day and measured with self-report or objective methods such as accelerometry [4, 5]. An alternative approach to operationalize SB would be to focus on a specific type of SB, such as total screen time [6]. The conceptual model by Biddle, Pearson, and Salmon [7] suggests that SB research should account for two types of SB, namely total sitting time and total screen time, because these two types of SB form different associations with health outcomes. Subtypes of SB may also be distinguished [8]: for example, total screen time can be divided further into TV watching, computer using, etc.

Recent studies on the prevalence of SB showed that children and adolescents (aged 5–18 years) as well as older adults (aged 60 ≥ years old) spend between 40% and 60% of their time sitting [9, 10]. High levels of SB may increase the risk of mortality and morbidity, independently of the levels of moderate-to-vigorous physical activity [11–13]. SB is associated with an increased risk of chronic physical health problems, including cardiovascular diseases, diabetes, and obesity [7, 14]. There is also a growing body of evidence suggesting associations between SB and mental health issues, including increased levels of anxiety [15, 16]. However, there are several open questions regarding associations between SB and anxiety symptoms, which could be clarified in an overarching synthesis of existing evidence. In particular, it is unclear how strong the SB—anxiety relationship is, if this association depends on the type of SB (e.g., total sitting time vs. total screen time), individual’s age or health status [16–18]. The present study attempts to clarify these issues.

Anxiety disorders rank among the most common psychiatric disorders with a lifetime global prevalence estimate of 7.3% (95% CI [4.8, 10.9%]) [19]. One in 14 people suffer from anxiety disorders around the world and one in nine (11.6, 95% CI [7.6, 17.7%]) will have an anxiety disorder in a given year [19]. Anxiety symptoms are common in diverse populations and feature excessive anxiety-linked emotional and behavioral disturbances as well as associated cognitive ideation [20]. Anxiety is a complex phenomenon involving state and trait components, defined as immediate emotional and somatic reactions to perceived demands and threats and stable inter-individual differences in tendencies to react in such a manner across demanding or threatening situations [21]. Anxiety symptoms occur across the lifespan, with anxiety disorders mostly developing before the age of 35 [22]. The median age of onset was established at 11 years old [23]. There are well-established associations between anxiety symptoms and an increased likelihood of metabolic diseases, cardiovascular incidents, cardiac mortality, diabetes, and stroke [24–27].

The links between SB and anxiety may be explained with physiological and psychological mechanisms. Experimental laboratory and real-life studies indicated that regular physical activity alters physiological responses to stressors which, in turn, affect anxiety levels [28–30]. Physiological pathways involve changes in central catecholamine systems and opioid mechanisms [30]. Additionally, serotonergic pathways may explain links between energy expenditure behaviors and anxiety symptoms. For example, activation of the 5-HT_2C_ receptor may elicit an anxiety-like response [31] whereas engaging in physical activity may decrease sensitivity of this receptor and thus reduce anxiety [32]. Psychosocial mechanisms linking anxiety and SB are suggested by the *displacement hypothesis*, proposing that SB displaces time available for other social and physical activities. It may be expected that several subtypes of screen-based SB, such as TV watching or playing video games, involve little social interaction or limit direct social interaction that influence mental health outcomes, including anxiety [33]. The displacement of physical activity with SB is associated with less favorable health outcomes [34]. Another psychosocial pathway linking SB and anxiety involves low self-esteem. People with low self-esteem may find physical and active social activities challenging, anxiety-evoking, and taxing, therefore they may be inclined to increase SB in leisure time [35].

Previous systematic reviews provided a preliminary synthesis of evidence for the relationship between SB and anxiety [18]. In particular, a review [18] of 9 studies concluded that the majority of research suggested a positive association between SB and anxiety. The number of studies has been growing in the recent years and a synthesis of findings, accounting for operationalization, measurement heterogeneity (e.g. different types of SB, such as total sitting time vs. total screen time vs. TV watching), and population heterogeneity (children/adolescents vs. adults; people from general population vs people with a chronic illness), is still missing. A meta-analytic approach may allow for a further synthesis of existing evidence and an investigation of the moderating role of the sources of heterogeneity.

Different types of SB may exert different effects on anxiety and anxiety symptoms, yet the evidence is inconclusive. For instance, a review of findings obtained in nine studies on the SB—anxiety symptoms association concluded that there is sufficient evidence for the link between total sitting time and anxiety, whereas the evidence for total screen time and the subtypes of screen time (TV watching, computer use) is inconsistent [18]. In contrast, findings from a recent meta-analysis suggested that the total sitting time is unrelated to anxiety, whereas total screen time as well as its subtype, TV watching, are related to anxiety [16]. However, the conclusions formed in previous reviews are preliminary as they are based on a very limited number of studies (e.g., *k* = 2 for total sitting time, *k* = 4 for total screen time, *k* = 3 for TV watching, [16]).

Theories, such as the socio-ecological approach, suggest associations between SB and socio-demographic factors, such as age [36–38]. For example, the Systems of Sedentary Behaviors framework [39], indicates that the link between SB and its psychosocial correlates (including anxiety or other mental health indicators) may be further moderated by age, with larger effects expected in older samples. Research on SB and its health outcomes usually targeted either samples from children/adolescent populations [40] or older-adult populations only [36, 41], thus the moderating effects of age remain unclear. Consequently, we investigated whether the strength of SB—anxiety relationship may depend on participants’ age group.

Additionally, although research on the SB—anxiety relationship was conducted among people with a chronic physical or mental illness, and in samples recruited from the general population, the effect of health status on SB—anxiety associations is still unclear [18]. A decline in health (or a chronic illness) may have an impact on the relationship between SB and anxiety symptoms [42]. Motl et al. [42] suggested that SB, illness-related physiological processes, and structural impairments are closely related: a combination of these factors may lead to a further decline in health and disability, but also to negative affective states [42]. As research usually focused either on people with a chronic illness or on the general population [43], the moderating effect of a chronic illness was rarely considered.

Our study aimed to summarize the evidence for the SB--anxiety relationship. We conducted a systematic review and meta-analysis in order to: (1) synthesize the associations between SB and anxiety symptoms and (2) examine if SB-- anxiety associations are moderated by the age group (children/adolescents vs. adults), participants’ health status (general population vs. people with a chronic physical or mental illness). Additionally, as the type of SB as well as SB operationalization and measurement may affect associations between SB and its health outcomes [44], we tested the moderating effects of the type of SB (total sitting time vs. total screen time), the subtype of total screen time (i.e., TV viewing vs. computer using vs. computer/video or console games playing), and SB measurement (self-report vs. objective measurement).

## Method

This study followed PRISMA guidelines [45] for systematic reviews and was registered with PROSPERO database (no. CRD42017068517).

### Search strategy

**Fig. 1:**
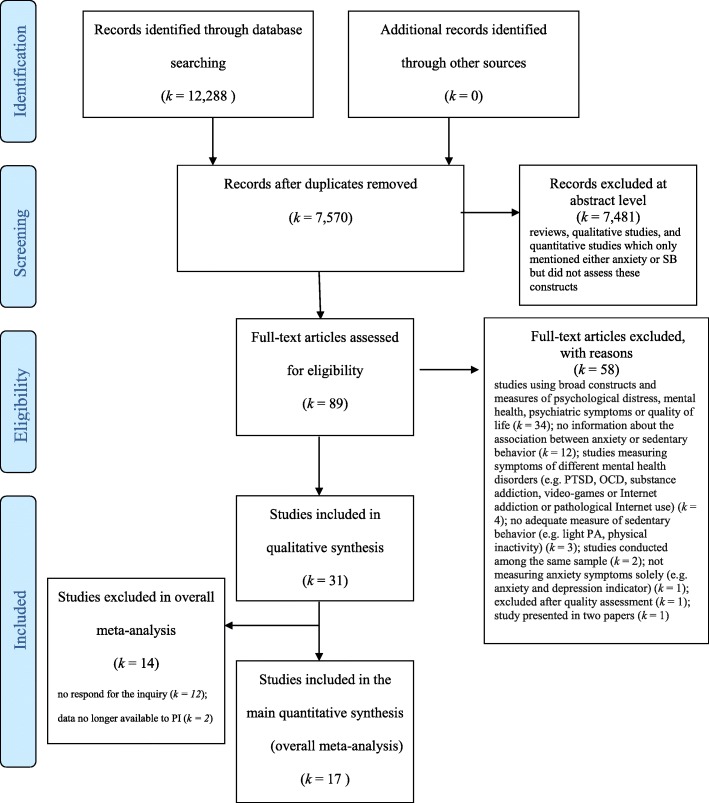
Flow-chart for search strategy

A systematic search of relevant studies published since the inception of the databases until April 2018 was conducted using: PsycINFO, PsycARTICLES, Academic Search Complete, ERIC, HealthSource: Nursing/Academic Edition and MEDLINE. To minimize bias, two researchers (BS and AB) independently performed an online search for peer-reviewed papers using the following combination of keywords: (‘sedentary beh*’ OR ‘sedentary*’ OR ‘sitting’ OR ‘screen*’ NOT ‘screening’ OR ‘screen time’ OR ‘screen-based’ OR ‘computer games’ OR ‘video games’ OR ‘television viewing’) AND (‘mental health’ OR ‘mental*’ OR ‘panic disorder’ OR ‘anxiet*’ OR ‘phobia’ OR ‘worry’ OR ‘worr*’). This strategy yielded 12,288 records (see Fig. 1; PRISMA flow chart). Studies were included if the keywords were present in either the title, or the abstract, or the original keywords of the paper. After identification of records through database searching, all duplicates (*k* = 7570) were removed. Additionally, the reference lists of selected studies and the related systematic review [18] were screened to identify relevant articles. Next, two independent researchers (BS and AB) read abstracts, keywords, and titles in order to establish if the paper reported an original study accounting for the associations between SB and anxiety. In case the abstract did not provide sufficient information to determine if the paper should be excluded, the researchers followed with reading the full-text. This strategy resulted in excluding *k* = 7481 entries: reviews, qualitative research, and quantitative studies which mentioned either anxiety or SB but did not assess these constructs. Next, 89 full-texts were assessed for eligibility (i.e., reporting a statistical test for the association between SB and anxiety). A total of 31 eligible studies were included and analyzed in the systematic review, with 17 studies included into the meta-analysis.

### Inclusion/exclusion criteria

Details of the selection processes are shown in Fig. 1. Overall, the selection process aimed at identifying any original studies determining the associations between SB and anxiety among children, adolescents, and adults of any age.

The main inclusion criteria were: (1) the relationship between SB and anxiety was reported, (2) SB was assessed with either self-report instruments (e.g., International Physical Activity Questionnaire; IPAQ) or an objective measure (e.g., accelerometer), (3) anxiety was measured quantitatively, without restrictions referring to the type of anxiety disorder or its stage (i.e. acute vs. chronic) or duration of anxiety symptoms, and (4) the quality assessment of the study, conducted with the tool by Kmet et al. [46], resulted in a score of at least 65% (for thresholds, see [46]). Only studies published in English in peer-reviewed journals were included. The decision to include only English-language publications was based on the results of a previous review of 303 meta-analyses [47] which showed that excluding trials published in languages other than English has little effect on the estimated of the effects. Publications in languages other than English were also likely to produce findings which may be more biased, as they included fewer participants, were more likely to produce significant results, and tended to have lower methodological quality than English-language publications [47].

The following exclusion criteria were applied: (1) studies measuring SB that occurred due to internet addiction or pathological internet use but excluding other types of SB, (2) studies accounting for anxiety as an undistinguishable subcomponent of broader constructs and measures, such as psychological distress or quality of life, (3) research with no adequate measure of SB (e.g., indicating only the presence/absence of a specific type of SB), and (4) research with populations with severe mobility limitations (and thus with extremely limited variability in sedentary time). In case the results from one and the same study were presented in two papers, the study with a larger sample or a more recent publication was selected.

### Data extraction and quality assessment procedures

Data extraction (see Table 1) was conducted independently by two researchers (BS and AB). Extracted data included details of SB and anxiety measurement, sample characteristics, and main findings of the original study. Selected statistical information and data necessary to conduct the quality evaluation were also retrieved. Any discrepancies during the process of data extraction and quality evaluation were resolved by a consensus method [48, 49], involving discussions between two researchers (BS, AB), and the third researcher (AL). In particular, in case of a discrepancy between two researchers (BS and AB), the third researcher (AL) retrieved respective data, conducted the quality evaluation independently, and led the discussion aiming at reaching a consensus. In case the data required to conduct meta-analysis were not included in the original paper, the research team attempted to contact authors via e-mail and requested the required data.

**Table 1:** Characteristics of analyzed original studies

Study	Country	Study design	Population	N (% of females)	Mean age (SD)	SB measure/indicator	Anxiety measure	Positive, negative or non-significant elation-ship between anxiety and the type of SB	Main effects/findings	Quality score (%)
Asfour et al., 2016 [59]	USA	Cross-sectional	Adolescents; (general population)	575 (45%)	13.8 ± 0.64 years	Time spent engaging in SB was calculated using five questions that inquired about time spent watching TV, playing video games, text messaging, Internet use and time spent on the telephone	Internalizing symptoms subscale of 112-item The Youth Self-Report [60]	0 for ST	Increased SB were associated with higher levels of externalizing symptoms (*β* = 2.03, SE = 0.32, *p* < .001), but not internalizing symptoms (*β* = 0.93, SE = 0.57, *p* = .1)	98
Asztalos et al., 2015 [61]^a^	Belgium	Cross-sectional	Adults (general population)	4344 (52%)	43.55 ± 11.05 years	Self-report - International Physical Activity Questionnaire (IPAQ) [62]	10-items for anxiety symptoms of Symptom Check List (SCL)(e.g. [63])	+ for sitting time	Sitting were positively associated with anxiety both in model adjusted for demographic (gender, age, education) (*β* = 0.037; *p* = .018) as well as adjusted for demographics and MVPA (*β* = 0.033; *p* = .038)	95
Bampton et al., 2015 [64]^a^	Canada	Cross-sectional	Older adults (≥ 55 yr) (general population)	358 (66%)	66.5 ± 8.0 years	Total and Domain Specific Measure of Sitting [5]	Generalized Anxiety Disorder scale (GAD-2) [65]	+ for sitting time (+ for high sedentary time/low resistance group)	Low sedentary time/high RT group reported lower anxiety symptoms compared to high sedentary time/low RT group (M_diff_ = − 0.67, *p* = .001). The low sedentary time/low RT group reported lower anxiety symptoms than high sedentary time/low RT group (M_diff_ = − 0.57, *p* = .018)	81
Cao et al., 2011 [66]	China	Cross-sectional	Adolescents (general population)	5003 (48%)	13.13 ± 0.97 years	Self-report to an open-ended question: how many hours per day, on average, the participants spent on the sedentary activities outside school hours on a usual weekday, as well as a weekend day (TV viewing, computer usage) [6]. ST was categorized as ≤2 h/d and > 2 h/d	41-item Screen for Child Anxiety Related Emotional Disorders (SCARED) [67]	+ for ST	High screen time (> 2 h/day) was a risk factor for anxiety symptoms both in crude model (*OR* = 1.39, 95% CI: [1.22, 1.60], *p* < .001) as well as in adjusted for gender, grade, family type, perceived socioeconomic status, BMI, fruit and vegetable or fizzy drinks intake (*OR* = 1.36, 95% CI: [1.18, 1.57], *p* < .001)	95
de Wit et al., 2011 [68]	Netherlands	Cross-sectional	Adults (general population)	2353 (65.45%)	41.2 ± 13.0 years	Self-report - the daily number of hours a person spent on watching TV or computer use in leisure time	Composite International Diagnostic Interview (CIDI, WHO ver. 2.1) [69]	+ for TV viewing; 0 for computer use	Anxiety was related to spending more time watching TV (*β* = 0.051, *p* < .05; *β* = 0.103, *p* < .001, respectively) but not to time of computer use (*β* = 0.001, *p* = .971; *β* = 0.036, *p* = .155, respectively) in leisure time	82
de Wit et al., 2015 [70]	Netherlands, Austria, Belgium, Ireland, Italy, Poland, Spain, UK, Denmark	Cross-sectional	Adult pregnant women (general population)	98 (100%)	31.6 ± 5.8 years	Actigraph GT3X, GT1M or Actitrainer accelerometer [activity were calculated as time spent sedentary (< 100 cpm)]	Pregnancy-related worries were measured with the 13-item Cambridge Worry Scale (CWS) [71]	0 for ASB	Pregnancy-related worries were not significantly associated with sedentary behaviors	100
Dillon et al., 2018 [72]	Ireland	Cross-sectional	Adults (general population)	397 (54%)	59.6 ± 5.5	A triaxial, GENEActiv accelerometer (ActivInsights Ltd., Kimbolton, Cambridgeshire, UK)	Anxiety subscale of the 14-item Hospital Anxiety and Depression Scale [73]	+ for ASB	Participants with moderate to severe anxiety symptoms had significantly more minutes of SB daily than those with low levels/ no symptoms of anxiety (*p* = .04)	100
Edwards & Loprinzi, 2016 [41]	USA	RCT	Adults (general population)	39 (59%)	Control: 22.08 ± 2.75; Intervention: 21.69 ± 2.71	GT9X accelerometers [21] and Digi-Walk SW-200 pedometer [74]	Overall Anxiety Severity and Impairment Scale (OASIS) [75]	+ for ASB	A statistically significant time x group interaction effect for OASIS scores *F*(1,37) = 11.13, *p* = .002). Mean and *SE* OASIS scores were significantly higher after the one-week sedentary behaviors-inducing intervention (M = 5.35, SE = 0 .86) compared to scores from before the intervention (M = 3.88, SE = 0.69) which means that an increase was observed in anxiety levels when participants increased their SB	89
Feng et al., 2014 [76]^a^	China	Cross-sectional	Young adults (general population)	1106 (43%)	18.90 ± 0.9 years	Self-report ST measured with one item: ‘How many hours per day do you spend on computer, including internet use, watching TV/video programs and playing games on a usual weekday and weekend day, respectively?’ The ST was categorized as ≤2 h/d and > 2 h/d	Self-rating anxiety scale (SAS) [77]	0 for ST	No statistically significant associations were found between ST and anxiety both for ≤2 h/day (*OR* = 1.52, 95% CI: [0.87, 2.64], *p* > .05) as well as for > 2 h/day ST levels	93
Gaskin et al., 2016 [78]	Australia	Cross-sectional	Prostate cancer survivors (chronic illness)	98 with complete data; 49 with no complete data	67.3 ± 8.0 with complete data; 62.1 ± 8.6 with no complete data	Hip-mounted ActiGraph GT1 M accelerometer (Pensacola, FL) units	Memorial Anxiety Scale for Prostate Cancer (MAX-PC) – three subcales (prostate cancer anxiety, prostate-specific antigen anxiety, fear of recurrence) and a total anxiety scale [79]	0 for ASB	Prostate cancer anxiety (*B* = 0.01, 95% CI: [− 0.03, 0.04], *p* = .78), prostate-specific antigen anxiety (*B* = 0.00, 95% CI: [− 0.00, 0.00], *p* = .96), fear of recurrence (*B* = − 0.01, 95% CI: [− 0.02, 0.01], *p* = .44) and a total anxiety (*B* = − 0.00, 95% CI: [− 0.05, 0.04], *p* = .94) were not significantly associated with SB	91
Gibson et al., 2017 [80]^a^	United Kingdom	Cross-sectional	Adults (general population)	42 (55%)	38.0 ± 11.5	ActivPAL mini, an inclinometer-based activity monitor	Anxiety subscale of The 14-item Hospital Anxiety and Depression Scale [73]	+ for ASB + for sitting time	Those with < 8 h of SB per day on weekdays had significantly lower levels of anxiety compared with those who were sitting > 8 h or > 10 h per day. The main effect for weekday sitting time on anxiety (*F*(1, 41) = 3.05, *p* = .040, *η*^*2*^ = 0.18)	77
Gunnell et al., 2016 [81]^a^	Canada	Longitudinal	Adolescents (general population)	1160 (61%) - Time 1	13.54 ± 1.12 years	Self-report questionnaire - 6-items querying how many hours per day subjects typically engaged in TV viewing/video game playing/computer use). The first 3 items assess ST during weekdays and the last 3 items inquired about weekend days	10-items Multidimensional Anxiety Scale for Children-10 (MASC-10) [82]	0 for ST	Initial symptoms of anxiety and ST did not predict change in ST and anxiety, respectively	95
Hiles et al., 2017 [83]	Netherlands	Longitudinal	Adults (general population)	2932 (66%) at baseline	41.9 ± 13.1	Self-report single question – sedentary behavior as an average hours of sitting on a weekday.	21-item Beck Anxiety Inventory (BAI; [84])	0 for anxiety- > sitting time change	Anxiety at the baseline did not predict SB at a 2-year follow up (*B* = 0.02, *p* = .561)	100
Janney et al., 2013 [85]^a^	USA	RCT	Adults with a diagnosis of schizophrenia/schizoaffective disorder with BMI > 27 (chronic illness)	46 (63%)	45.6 ± 9.8 years	ActiGraph AM-7164 accelerometer (ActiGraph, Ft. Walton Beach, FL). Sedentary was established as ≤100 cpm	PANSS (one item for anxiety) [86]	0 for ASB	No association was observed between SB and PANSS psychiatric symptoms (PANSS or subscale: *p* ≥ .59). There were no significant associations for the PANSS questions asking about anxiety (*r*_*s*_ = .22, *p* = .15 for sedentary minutes and *r*_*s*_ = .15, *p* = .32 for percentage of sedentary time)	89
Kovess-Mastefy et al., 2015 [87]	German, Netherlands, Lithuania, Romania, Bulgaria, Turkey	Cross-sectional	Schoolchildren (general population)	3184	8.72 years	Parents were asked how long their child spends playing video games on average during the week. Low video game use was defined as 0–60 min per week; moderate use was defined as 61–300 min, and high use was > 300 min.	GAD indexes of self-reported mental health computerized cartoon-like assessment tool ‘Dominic Interactive’ for children [88]	0 for video game playing	Playing video games (1–5, and 5+ vs. 1 or less h) was not associated with GAD (*OR* = 1.08, 95% CI: [0.69, 1.7]; *OR* = 0.95, 95% CI: [0.53, 1.69], *p* > .05, respectively)	91
Kroeders et al., 2013 [89]^a^	Australia	Cross-sectional	Stroke patients (chronic illness)	19 (53%)	66.2 ± 19.3 years	PAL2 electronic device - dual axis accelerometer	Anxiety subscale from Irritability, Depression and Anxiety (IDA) scale [90]	- for sitting time	Patients with anxiety symptoms compared with those without symptoms tended to spend more time lying (mean 64% vs. 43%), less time sitting (mean 33% vs. 51%), and less time standing or walking (mean 2% vs. 6%). The difference between these groups in time spent lying bordered on significance (*t*(17) = − 2.0; *p* = .06)	76
Liu et al., 2016 [91]^a^	China	Cross-sectional	Secondary school pupils/adolescents (general population)	13,659 (49%)	15.18 ± 1.89 years	The Youth Risk Behavior Survey (YRBS) questionnaire [92] ‘How many hours do you watch television or play VG/CU (including activities such as Nintendo, Game box, Xbox, computer games, and the internet) on a typical school day?’ The ST was categorized as: non-ST (0 h/day), occasional ST (> 2 h/day), moderate ST (> 1 to ≤2 h/day), high ST (> 2 h/day)	The Multidimensional Anxiety Scale for Children (MASC) [82, 93]	+ for TV viewing; + for VG/CU time	More than 2 h per school day of TV watching was associated with higher risk of anxiety in boys (*OR* = 1.43, 95% CI: [1.05, 1.95]) compared with no TV exposure. School day with high VG/CU time (> 2 h) was associated with higher risks of anxiety in boys (*OR* = 1.40, 95% CI: [1.061.86]) compared with no VG/CU	98
Maras et al., 2015 [94]^a^	Canada	Cross-sectional	Adolescents (general population)	2482 (58%)	14.10 ± 1.57 years	The Leisure-Time Sedentary Activities 6-item questionnaire measured how many hours per day subjects typically engage in: TV viewing/video game playing, computer use). The first 3 items assess screen-based activities during a typical weekday and the last 3 items screen time accrued on a typical weekend day.	The Multidimensional Anxiety Scale for Children-10 (MASC-10)	+ for ST (hours per day of TV viewing; recreational computer use; video games); + for video game playing; 0 for TV viewing; 0 for computer	Duration of screen time (*β* = 0.07, *p* < .001) and VG playing (*β* = 0.11, *p* < .001) was associated with severity of anxiety.	93
Mesquita et al., 2017 [95]	Netherlands	Prospective observational study	Adults with COPD before/after pulmonary rehabilitation (chronic illness)	90 (40%)	67.0 ± 8.0	CIRO activity monitor (CAM or the MOX Activity Monitor (Maastricht Instruments B.V in Maastricht, the Netherlands)	Anxiety subscale of the 14-item Hospital Anxiety and Depression Scale [73]	0 for anxiety- > ASB change	Baseline anxiety levels were unrelated to changes in minutes of SB (pre-post rehabilitation, Spearman *R* = −.08	95
Opdenacker, & Boen, 2008 [96]^a^	Belgium	Longitudinal	Adults (general population)	66	2 groups, aged *M* = 38.8 ± 11.4 years; and 39.9 ± 9.9 years	Self-report - International Physical Activity Questionnaire (IPAQ) [62]	The Spielberger state-trait anxiety inventory (STAI) [21]	0 for sitting time	Sitting time was not associated with anxiety (*r* = 0.46, *p* = .623).	85
Padmapriya et al., 2016 [97]^a^	Singapore	Cross-sectional	Pregnant women (general population)	257 with state anxiety symptoms; 270 with trait anxiety symptoms	29.5 ± 5.7 with state anxiety symptoms; 29.5 ± 5.6 with trait anxiety symptoms	Two categories of self-reported total sitting time per day and TV viewing time per day: < 7 h of total sitting time per day and < 2 h of TV viewing time per day were identified as lower tertiles; ≥7 h of total sitting per day and ≥ 2 h of TV viewing time per day were defined as higher total sitting time and TV time	The Spielberger state-trait anxiety inventory (STAI)	+ for TV viewing	Women with higher TV viewing time had higher state anxiety compared to women with lower TV viewing time in crude analysis (*OR* = 1.56, 95% CI: [1.14, 2.14], *p* = .006)	95
Park et al., 2017 [98]^a^	United Kingdom	Cross-sectional	Older adults (residents from assisted living facilities), (chronic illness)	87	77.5 ± 8.2	Accelerometers (GT3X+, WGT3X-BT; ActiGraph (Pensacola, FL, USA)	Anxiety subscale of the 14-item Hospital Anxiety and Depression Scale [73]	- for ASB	SB was negatively associated with anxiety (*r* = −.39, *p* < .01)	95
Rebar et al., 2014 [99]^a^	Australia	Cross-sectional	Adults (general population)	1104 (55%)	58 (range 48–66)	10-item Workforce Sitting Questionnaire [100]	Anxiety subscale from 21-item Depression, Anxiety, and Stress Scale (DASS-21) [101]	+ for overall sitting time + for computer sitting 0 for leisure/work/TV sitting	Overall sitting time (*b* = 0.03, *p* < .05) and computer use (*b* = 0.03, *p* < .05) were significantly associated with anxiety. Leisure (*b* = 0.01, *p* > .05), work (*b* = 0.02, *p* > .05), and TV (*b* = 0.00, *p* > .05) were not associated with anxiety	100
Sanchez-Villegas et al., 2008 [102]	Spain, USA	Longitudinal	Adults (general population)	10,381	27 years	Self-report sedentary index: hours per week spent on watching television and/or using computer.	Self-reported anxiety: ‘Have you ever been diagnosed of anxiety by a health professional?’ - classified as an incident case of anxiety	0 for ST	There was no significant association between the sedentary index and anxiety risk (*p* = .17)	89
Straker et al., 2013 [103]^a^	Australia	Longitudinal	Adolescents (general population)	643 (54%)	14.0 ± 0.2 years	Screen Based Media – Self-report recall electronic diary/questionnaire MARCA - clusters: C1. instrumental computer gamers; C2. multi-modal e-gamers; C3. computer e-gamers [104]	Internalizing symptoms index of 112-item the Youth Self-Report [59]	+ for game playing	C1 males reported less internalizing behavioral problems than C2 (difference − 1.7, 95% CI: [− 3.5, 0.1], *p* = .065) or C3 males (difference − 2.6, 95% CI: [− 4.9, − 0.3], *p* = .027)	91
Teychenne & Hinkley, 2016 [105]^a^	Australia	Cross-sectional	Adult women with children aged 2–5 years (general population)	575 (100%)	37.18 ± 4.62 years	Self-report –of women’s own activities, including time spent on TV/DVD/video viewing, computer/e-games/hand held device use on a typical weekday and weekend day	7-items related to symptoms of anxiety experienced in the past week: a subscale (HADS-A) of the Hospital Anxiety and Depression Scale (HADS) [106]	+ for computer /device use + for total ST; 0 for TV viewing	TV viewing was not associated with anxiety symptoms (*B* = 0.109, *p* = .188) but computer/device use (*B* = 0.212, *p* = .011) and overall screen time (*B* = 0.109, *p* = .025) were positively associated with heightened anxiety symptoms in models adjusted for key demographic and behavioral covariates (including MVPA)	100
Uijtdewilligen et al., 2011 [107]	Netherlands	Longitudinal	Adolescents (general population)	217 (58%)	M: 13.0 ± 0.6 years; F: 12.9 ± 0.6 years	Acti-Graph accelerometers (Model GT1M, ActiGraph, LLC, Fort Walton Beach, FL)	Facilitating anxiety index of Achievement Motivation Test (AMT) [108]	+ for ASB	In males, a higher score on facilitating anxiety (*B* = 5.13, 95% CI: [0.08, 10.19], *p* < .05) was associated with more minutes spent sedentary in adulthood.	88
Vallance et al., 2015 [109]^a^	Canada; Australia	Cross-sectional	Adults (general population)	197 (overall 180–45%)	64.3 ± 10.3	Acitgraph GT3X+ accelerometer (Actigraph, LLC, Pensacola, FL)	Spielberger’s State Anxiety Inventory (STAI) - 10 items	0 for ASB	No significant associations emerged for sedentary time and psychological health outcomes (including anxiety) [Wilks’ λ = 0.956, *F*(9382.3) = 0.788, *p* = .628]	93
van Roekel et al., 2016 [110]^a^	Netherlands	Cross-sectional	Adults treated for stage I–III colorectal cancer (chronic illness)	145 (37.2%)	70.0 ± 8.7 years	The triaxial MOX activity monitor (MMOXX1, upgraded version of the CAM monitor)	Anxiety subscale of the 14-item Hospital Anxiety and Depression Scale [73]	0 for ASB (0 for sedentary per 1 h/day)	Substituting sedentary time with physical activity was not significantly associated with lower anxiety (*β* = −0.7, 95% CI: [− 1.7, 0.3])	95
Vancampfort et al., 2018 [111]	China, Ghana, India, Mexico, Russia, South Africa	Cross-sectional	Adults (general population)	42,469 (50.1%)	43.8 ± 14.4	Self-report sitting time – total time usually spent (expressed in minutes per day) sitting or reclining including at work, at home, getting to and from places, or with friends (e.g., sitting at a desk, sitting with friends, travelling in car, bus, train, reading, playing cards or watching television)	Self-reported anxiety by the question ‘Overall in the past 30 days, how much of a problem did you have with worry or anxiety’ with response alternatives: none, mild, moderate, severe, extreme. Those who answered severe or extreme were considered to have anxiety	+ for sitting time	Anxiety was significantly associated with higher mean time spent sitting (*b* = 24.16, 95% CI: [6.95, 41.38], *p* < .01)	100
Wu et al., 2015 [112]	China	Cross-sectional	Adults (general population)	4747 (58.4%) - 16.3% with anxiety	19.2 ± 1.41 years	Self-report screen time measured with one item: ‘How many hours per day do you spend on the computer (including playing video or computer games or computer for something) and watching TV/video programs on a usual weekday and weekend day, respectively?’ ST was categorized as ≤2 h/d and > 2 h/d	Self-rating anxiety scale (SAS) [77]	+ for ST	High screen time > 2 h/day (*OR* = 1.38, 95% CI: [1.15, 1.65], *p* < .001) was significantly positively associated with anxiety in crude model as well as in a model adjusted for gender, age, residential background, BMI, perceived family economy and perceived study burden (*OR* = 1.49, 95% CI: [1.24, 1.79], *p* < 0.001)	98

*ASB* accelerometry measured sedentary behaviors, *SB* sedentary behaviors (three article which provided broad definition of obtained sedentary behaviors index), *ST* total screen time, *MVPA* moderate-to-vigorous physical activity, *RT* resistance training, *VG* video games, *CU* computer use, *GAD* generalized anxiety disorder

^a^studies included into meta-analysis

To evaluate the quality of identified studies, a tool by Kmet et al. [46] was applied. This tool for quality determination addresses the following criteria: the clarity of research objectives; the description of study design, participants, measures, randomizations, blinding, the selection of outcomes, rationale for the sample size and analytic method, estimates of variance reported for the main results/outcomes, a control of analyses for confounding effects; reporting results in sufficient detail. Each component was rated using a 3-point response scale (2 points for ‘yes’, 1 point for ‘partial’, 0 points for ‘no’). If the criterion was not applicable for a study, then its score was excluded from the computation of the overall score. The cut-point for the inclusion was 65% (indicating a moderate-to-high quality) of the potential maximum score. The 65% threshold was chosen from five possible cut-off points (75, 70, 65, 60, and 55%) proposed by Kmet et al. [46], who defined cut-offs as ranging from conservative (75%) to liberal (55%), with 65% representing the moderate cut-off threshold. Overall, the quality of 32 studies was evaluated; one study did not meet the 65% threshold and was excluded from analyses. Thus, a total of 31 relevant studies met the eligibility criteria and were systematically reviewed. Additionally, 17 out of 31 studies reported coefficients for SB--anxiety associations. These studies were included into the meta-analysis. The concordance coefficients for quality assessment were moderate (all Kappas ≥ .65, *p* < .001). The overall scores are presented in Table 1.

### Coding

All stages of data coding were conducted independently by two researchers (BS and AB). Next, the third researcher (AL), compared the coding agreed by two researchers (BS and AB) with the data reported in the original studies. This check was conducted for all included studies (100%).

For the purpose of this review, SB was defined as any waking behavior characterized by an energy expenditure ≤1.5 METs while in a sitting or reclining posture (Sedentary Behavior Research Network [50]). Where applicable, SB was coded into two broad types proposed by Biddle et al. [7]: (1) total sitting time and (2) total screen time. Additionally, as proposed by the Sedentary Behavior Research Network [8], subtypes of screen-based behaviors were distinguished: (3) TV viewing; (4) any computer use, (5) computer/video/console games playing.

Total sitting time was coded as the amount of time spent sitting/reclining during any leisure activities including sitting at work, reading, TV viewing, sitting at desk, and transport time, etc. Total screen time was coded as the amount of time spent sitting in front of a screen (including TV watching, using mobile devices, internet, computers/game consoles, etc.). For the purpose of the meta-analysis, the following three specific subtypes of screen time were coded: (1) TV viewing, (2) any computer use, (3) computer/video/console games playing (see Additional file 1). A similar approach to SB categorization was used in previous systematic reviews (e.g., [18]) which analyzed total screen time, as well as the subtypes of screen time, such as computer/internet use and TV viewing.

Next, data referring to SB were coded depending on the measurement methods. SB was coded as objectively measured if SB was assessed with accelerometers, pedometers, and position activity electronic loggers (see Table 1). Self-report measurements of SB included questionnaires and structured interview methods (see Table 1).

Anxiety symptoms were defined as either presence or intensity of symptoms of the most frequent subtypes of anxiety disorders, that is generalized anxiety disorder, phobias, separation anxiety disorder, panic disorder symptoms [22], or other non-clinical anxiety-related reactions (e.g., the level of general anxiety). The applied measures of anxiety symptoms included questionnaires and structured interviews (see Table 1). These measures were used to assess different types of anxiety symptoms such as: phobic anxiety, agoraphobia, panic disorder, generalized anxiety, separation anxiety, social anxiety, facilitating anxiety, debilitating anxiety, trait anxiety, state anxiety, internalizing behavior (anxious-depressed, withdrawn, somatic), prostate cancer anxiety, prostate-specific antigen anxiety, fear of recurrence, or incident cases of anxiety. Several self-report instruments applied in original studies have been established as screening tools with a validated threshold indicating the presence of an anxiety-related diagnosis (e.g., MASC, OASIS, SCARED; see Table 1).

Studies were coded as referring to ‘children and adolescents’ or ‘adults’ if the mean age of participants was < 18 or ≥ 18 years old, respectively. There were no studies combining children, adolescents, and adult samples.

Next, studies were coded with respect to the health status of participants. The health status was coded as ‘general population’ if the sample was drawn from a non-clinical, general population and if there were no inclusion criteria regarding the presence of a chronic illness (either physical or mental). Two studies, enrolling samples drawn from a general population of healthy pregnant women, were also coded as ‘general population’. The health status was coded as ‘with a chronic illness’ if the sample was drawn from a population with a diagnosed chronic physical illness (e.g., participants diagnosed with colorectal cancer) or a mental illness (schizophrenia or schizoaffective disorder). There were no studies that examined samples combining ‘general population’ and ‘chronic illness’ categories.

### Methods of data synthesis and data analysis

Two methods of data synthesis were applied. The use of two methods of data synthesis allows for a cross-check between meta-analytical findings (obtained with a more robust and established method, but conducted with a smaller number of studies) and a synthesis of data in a systematic review (based on a-priori selected thresholds; not accounting for the heterogeneity of studies).

To synthesize systematic review-allocated data from 31 studies, we applied a synthesis strategy based on a-priori selected thresholds, accounting for the proportion of significant associations across included studies (for previous use of this strategy see Boberska et al. [51]; Luszczynska et al. [52]). Data indicating that the association between an index of SB and an index of anxiety symptoms was significant were retrieved from the original research and defined as ‘a relationship unit’. Subsequently, depending on the direction of the association each unit was coded as ‘+’ or ‘-’ if a significant positive association between SB and anxiety was reported, and ‘0’ if the association was not significant. To summarize findings of the original studies, evidence ratings were coded as: (1) ‘showing corroborating evidence’ if 60–100% of the original studies supported the association; or (2) ‘showing preliminary support’ if 50–59% of the studies supported the association [53].

The indicators of the associations between SB and anxiety symptoms were retrieved (in particular, the correlation coefficients, regression coefficients, path coefficients, odds ratios etc.). In case of experimental studies, the coefficient representing the main effect of a manipulation was used in the data analysis. In case of studies comparing groups with different levels of SB, data regarding levels of anxiety symptoms in each group were obtained and compared. In case of longitudinal studies, coefficients representing the associations between the baseline and the latest available follow-up were included into analysis.

Overall, 25 studies yielded 1 association coefficient each, 3 studies yielded 2 coefficients, 2 studies yielded 3 coefficients, and 1 study yielded 4 coefficients. Two or more coefficients were obtained if the original study provided indicators of associations for more than one type of SB (e.g., 1 coefficient for total sitting time and 1 for TV watching). Thus, a total of 41 coefficients from 31 studies were included into the data synthesis.

In order to calculate the estimates of the average effects, heterogeneity, and the effects of the moderators, data obtained from 17 original studies were meta-analyzed using the Comprehensive Meta-Analysis software (version 2.2) [54], which is the most extensively used meta-analytic software [55]. The meta-analysis was conducted for original research providing bivariate association coefficients obtained in an equation without covariates. Pearson’s correlation was used as the effect size indicator (see Additional file 1). Correlations were synthesized to form the cumulative effect size by transforming into Fisher’s *z* according to the procedures described by Borenstein et al. [56]. If a publication did not provide the respective coefficients, the authors were contacted by e-mail with a query to provide *r* coefficients. Seven correlation coefficients were obtained from original publications; 10 correlation coefficients were obtained from authors directly.

**Fig. 2:**
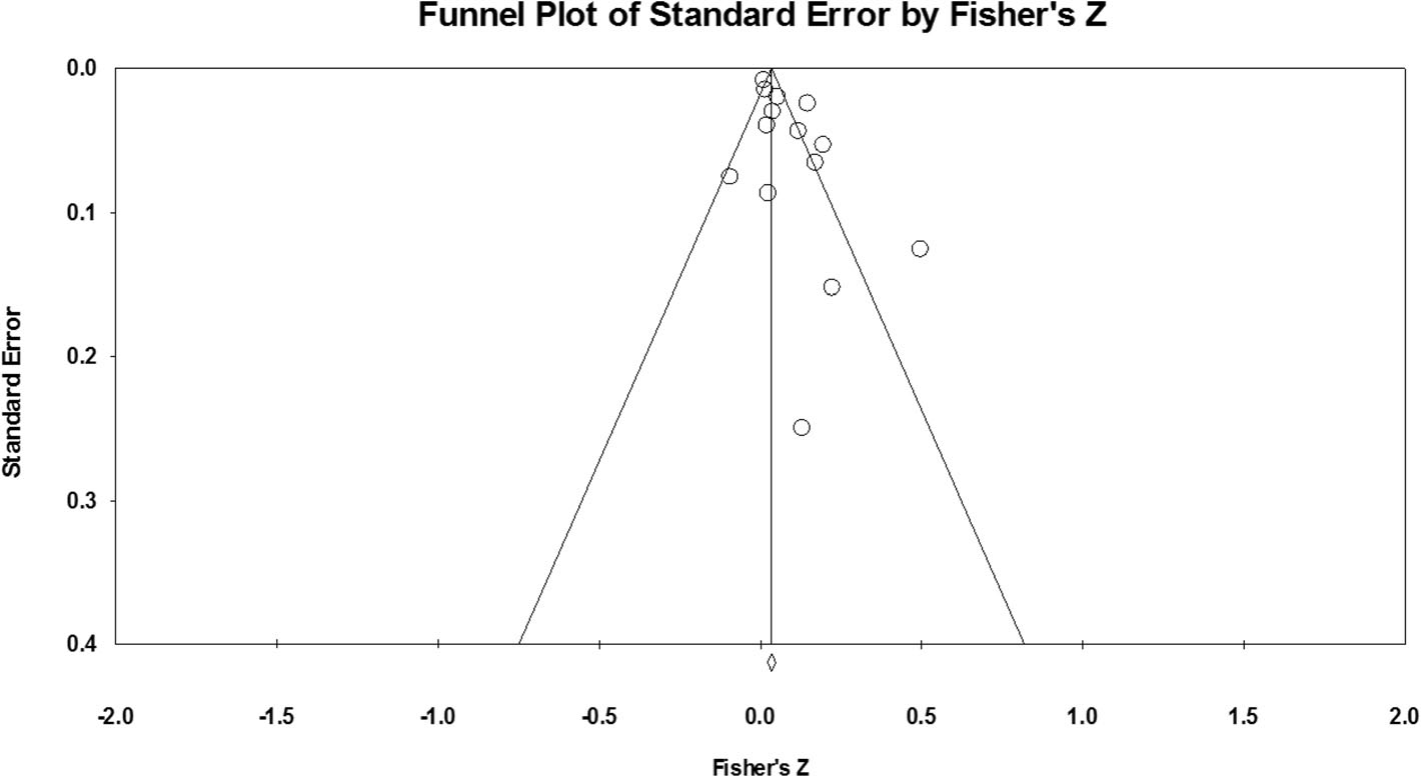
The funnel plot of standard errors by Fisher’s z

A random-effects model was used to calculate the estimate of the population effect size. To investigate the asymmetry which may be caused by publication bias, the funnel plot for 17 studies (see Fig. 2) was screened and Egger’s test was conducted. Statistical analyses followed the procedure described by Hunter and Schmidt [57]. First, an overall effect was determined for all original studies included in the meta-analysis. Next, we performed moderation analyses to investigate if there were differences in estimated effects depending on participants’ age, health status, and the type of sedentary behavior. The moderation analyses were conducted if the number of respective subgroups was *k* ≥ 2. Only one study included self-reports of children, therefore research enrolling children and adolescents were combined into one subgroup.

To test the effects of the moderators, the estimate of the effect size was calculated for each level of a moderator. Next, group mean effect sizes were compared using the *Qʙ* statistic. *Qʙ* is used as an omnibus test for detecting between-group differences [58]. A significant *Qʙ* value indicates that estimates of the average effect differ significantly for ≥2 levels of the moderator.

## Results

### Search results

We identified a total of *k* = 31 studies eligible for inclusion into a systematic review and 17 studies eligible for inclusion in the meta-analysis. Details of the search process are presented in Fig. 1. Data retrieved from the original studies are summarized in Table 1.

### A synthesis of findings from studies included into the systematic review

A total of 99,192 participants were enrolled in all 31 studies with sample sizes ranging from 19 to 42,469 participants. Participants’ age ranged from 6 to 70+. Six studies (20%) included adolescents, 1 (3%) included children and adolescents, and 1 (3%) enrolled children only. Twenty-three studies (74%) were conducted in adult populations. Overall, 25 studies (81%) involved general population samples, whereas 6 (19%) enrolled adults with a chronic mental illness (i.e., schizophrenia or schizoaffective disorder) or physical illness (stroke, colorectal cancer, chronic obstructive pulmonary disease, cardiovascular diseases, musculoskeletal diseases, diabetes, lung disease, obesity, prostate cancer survivors). Across original studies, the majority (*k* = 22, 71%) applied cross-sectional designs, 7 (23%) were of correlational longitudinal designs, 2 (6%) studies were experimental. Regarding the assessment of SB, the majority of studies (*k* = 19, 61%), relied on self-report whereas *k* = 12 (39%) studies used objective methods. Assessments of anxiety symptoms were mostly self-report (*k* = 29; 94%) whereas in *k* = 2 (6%) studies anxiety symptoms were assessed with an interview. Original studies were conducted in 24 different countries, across Europe, North America, Asia, Australia, and Africa. The quality score assessment of included studies ranged between 76 and 100% (see Table 1).

Across 41 associations obtained from original studies, 21 (51%) indicated that higher levels of SB were associated with higher levels of anxiety (Table 2). Fifty-eight percent (7 out of 12) of obtained associations among children/adolescent samples indicated corroborating evidence for a positive link between SB and anxiety. For adults, 61% (14 out of 23) of associations were positive. Evidence supporting the SB--anxiety relationship among adults from the general population was indicated by 14 (48%) out of 29 coefficients reported in original studies. There was no support for the SB—anxiety symptoms association in studies enrolling adults with chronic illnesses (0 out of 6 obtained associations). No extracted study with children/adolescents was conducted among participants with a chronic illness.

**Table 2:** Associations between SB and anxiety symptoms among children/adolescents and adults

Sample/Category of SB	Accelerometry measured sedentary behaviors)	Total sitting time	Total screen time	TV viewing	Computer use/internet use/video game playing	Proportion of significant positive associations across studies (regardless of the category of SB)	Proportion of significant positive associations: Adults vs. children/adolescents (regardless of the category of SB)	Proportion of significant positive associations: general population vs. with chronic illness (regardless of the category of SB)
Adults								
General population	+ (Edwards & Loprinzi, 2016; Gibson et al. 2017; Dillon et al., 2018) [41, 72, 80]; 0 (de Wit et al., 2015; Vallance et al., 2015) [70, 109]	+ (Asztalos et al., 2015; Bampton et al., 2015; Rebar et al., 2014; Gibson et al., 2017; Vancampfort et al., 2018) [61, 64, 80, 99, 111]; 0 (Opdenacker, & Boen, 2008; Hiles et al., 2017) [83, 96]	+ (Teychenne & Hinkley, 2016; Wu et al., 2015) [105, 112]; 0 (Feng et al., 2014; Sanchez-Villegas et al., 2008) [76, 102]	+ (de Wit et al., 2011; Padmapriya et al., 2016) [68, 99]; 0 (Rebar et al., 2014; Teychenne & Hinkley, 2016) [99, 105]	+ (Rebar et al., 2014; Teychenne & Hinkley, 2016) [99, 105]; 0 (de Wit et al., 2011) [68]	Adults; general population: 14 (61%) in 23 associations	Total adults: 14 (48%) in 29 associations	Total for general population (children/adolescents vs. adults) 21 (60%) in 35 associations
With a chronic mental or physical illness	0 (van Roekel et al., 2016; Janney et al., 2013; Mesquita et al., 2017; Gaskin et al., 2016) [78, 85, 95, 110]; - (Park et al., 2017) [98]	- (Kroeders et al., 2013) [89]						
Children and adolescents (aged < 18)								
General population	+ (Uijtdewilligen et al., 2011) [107]		+ (Cao et al., 2011; Maras et al., 2015) [66, 94]; 0 (Asfour et al., 2016; Gunnell et al., 2016) [59, 81]	+ (Liu et al., 2016) [91]; 0 (Maras et al., 2015) [94]	+ (Straker et al., 2013; Maras et al., 2015; Liu et al., 2016) [91, 94, 103]; 0 (Kovess-Mastefy et al., 2015; Maras et al., 2015) [87, 94]	Children/Adolescents; general population: 7 (58%) in 12 associations	Total children/adolescents: 7 (58%) in 12 associations	
With a chronic mental or physical illness								
Proportion of significant positive associations across studies (regardless age/ health status)	4 (36%) of 11 associations	6 (67%) of 9 associations	4 (50%) in 8 associations	3 (50%) of 6 associations	5 (63%) in 8 associations	21 (51%) in 41 associations		

^+^a significant positive association between sedentary behavior and anxiety symptoms

^0^not significant association between sedentary behavior and anxiety symptoms

^-^a significant negative association between sedentary behavior and anxiety

Additional analyses focused on the type of SB (total sitting time vs. screen time) and the subtypes of screen time (TV viewing vs. computer use/internet use/video game playing) and its measurement (objective vs. self-report) (Table 2). Corroborating evidence for a positive association between SB and anxiety symptoms was found for: self-reported sitting time (6 out of 9 original studies accounting for this index; 67%); computer use/internet use/video game playing (5 out of 8 studies; 63%). However, only 3 out of 6 original studies (50%) and 4 out of 8 studies (50%) indicated a positive relationship between TV viewing and anxiety and total screen time and anxiety, respectively. Finally, across studies focusing on objectively measured total sitting time and anxiety, only 4 out of 11 (36%) yielded positive associations suggesting that higher levels of SB were related to higher levels of anxiety symptoms.

### The meta-analytic synthesis of findings

A total of 27,443 participants were enrolled in 17 original studies (see Table 3), with sample sizes ranging from 19 to 13,659 (*M* = 1614) participants, and 64.18% women participating. One study did not provide the distribution for gender. Mean age of the participants was 41.91 years old (*SD* = 22.05), ranging from 13.54 to 77.5. Five studies were conducted among children/adolescents from the general population (*N* = 19,050, mean age = 15.34, *SD* = 2.62). Seven studies enrolled adults (*N* = 7125, mean age = 51.44, *SD* = 16.03) without any clinical illness reported (two studies enrolled pregnant women). Five studies enrolled adults with a chronic physical or mental illness (participants: *N* = 475, mean age = 64.72, *SD* = 11.82), such as cardiovascular diseases, musculoskeletal diseases, diabetes, lung disease, obesity, schizophrenia or schizoaffective disorders, stroke, colon or colorectal cancer. Thirteen studies (76%) had a cross-sectional design, 3 (18%) used a longitudinal correlational design, and 1 (6%) applied an experimental longitudinal design.

**Table 3:** Results of meta-analysis and moderation analysis of the association between SB and anxiety symptoms

	Estimate of the average effect	Range of correlation coefficients retrieved from original studies	95% CI for the estimate of the average effect	*N*	*K*	Heterogeneity				Test for moderating effects	
						*Q*	*I* ^*2*^ *%*	*Tau*	*Tau* ^*2*^	*Q*_*B*_	*P*
Overall effect	.093	.01; .46	[.05; .13]	26,204^b^	17	77.04 *p* < .001	79.23	.06	.003		
Moderators effects for overall effect and levels of respective moderators											
Age group										2.97	.085
Children/adolescents	.05	.01; .17	[−.01; .11]	17,873	5						
Adults (over 18)	.12	.02; .46	[.06; .17]	7868	7						
Health status										0.05	.820
Adults with a chronic physical or mental illness	.16	.03; .39	[.03; .30]	463	5						
Adults from the general population	.15	.02; .46	[.05; .23]	6990	6						
The type of measurement of sedentary behaviors										1.08	.299
Objective	.14	.03; .39	[.04; .24]	505	6						
Self-report	.08	.01; .46	[.04; .12]	25,699	11						
The type of SB										2.21	.137
Total sitting time	.12	.02; .46	[.06; .19]	7298	5						
Total screen time	.06	.01; .17	[.00; 11]	18,401	6						
The sub-type of screen use-related behaviors										5.04^a^	.080
Computer using	**.12**	.10; .14	[.05; .18]	2183	2						
Computer/video games playing	**.02**	.02; .03	[−.03; .08]	15,896	2						
TV viewing	.05	−.001; .10	[.01; .09]	16,475	4						

*Note*. ^a^ - Two-group comparisons revealed that effect sizes marked with bold were significantly different

^b^ - data from 26,204 participants were included from the total of 27,443 who were enrolled across 17 studies. The difference between the number of participants in analyses vs. the original study samples occurred as in cases the coefficients provided by authors in response to our inquiry were based on a smaller *N* than *N* reported in the publication

An inspection of the funnel plot and the values of the Egger test (intercept: 2.11; *p* < 0.01) indicated that the smaller studies tended to have better test performance. These findings suggest a likelihood of a publication bias.

Table 3 displays the results of the meta-analysis, including the estimates of the average effects and moderator analyses. The estimate of the overall average effect for the association between indicators of SB and anxiety symptoms was significant and small with weighted *r* = .093, 95% CI [.055, .130], *p* < .001, suggesting that higher levels of SB are associated with higher levels of anxiety symptoms. Table 3 displays the estimates of heterogeneity, *Tau*^*2*^, *Tau,* and *I*^2^ [113]. To demonstrate how much an effect might vary across different populations, prediction intervals were calculated with *Tau* (*τ* = .058), using an approach described by Borenstein et al. [114]. Based on these findings, it can be expected that in 95% of different populations, the true correlation will fall in the approximate range of −.030 to .214.

Moderation analyses were performed to address this dispersion and to take into account the estimates of obtained heterogeneity. First, we tested if age group (children/adolescents vs. adults) would moderate the association between SB and anxiety (Table 3). Two types of studies were compared: (1) enrolling children/adolescents (*k* = 5 samples) and (2) enrolling adults aged over 18 years (*k* = 7). As there were no studies with children/adolescents with a chronic illness, studies conducted among adults with chronic illnesses (*k* = 6) were excluded from the analysis to avoid the effect of a potentially confounding factor, the presence of a chronic illness. The comparison yielded a statistical trend for a difference between obtained estimates (*p* = .085), indicating that the associations tended to be stronger in adults, compared to associations obtained for children/adolescents. To test the moderating effect of health status (adults from the general population vs. adults with a chronic mental of physical illness) two types of studies were compared: (1) enrolling adults from the general population (*k* = 5); and (2) enrolling adults with a chronic mental or physical illness (*k* = 6). Results indicated no differences between the average effects obtained for the two groups (*p* = .820).

The moderating effect of the type of SB was investigated with a comparison of two subgroups: (1) studies which investigated total screen time (*k* = 6), and (2) studies which investigated total sitting time (*k* = 5). Only studies that used self-report measures were included into this moderation analysis (total *k* = 11). Results did not show any significant differences between the estimates of average effects obtained for the two types of SB (*p* = .137).

Next, we conducted the moderation analysis comparing associations between anxiety symptoms and three subtypes of screen time (TV viewing vs. computer using vs. computer/video/console games playing). This analysis was performed with data obtained from 4 studies, all of which accounted for ≥2 types of SB (e.g., TV viewing and computer using separately). In particular, 8 coefficients were included: 4 coefficients were obtained for TV viewing, 2 for computer using, and 3 for computer/video/console games playing. Thus, we compared associations obtained for: (1) indicators of time spent watching TV (*k* = 3), (2) indicators of time spent using a computer (*k* = 2), and (3) indicators of time spent on playing computer/video games (*k* = 2). The comparison of all three types of SB yielded a statistical trend for a difference (*p* = .080). The following two-group comparisons indicated that the average effects for computer/video/console games playing were significantly smaller than the effects for computer using (*p* < .001). There were no significant differences in the effects of TV viewing compared to computer/video/console games playing (*p* = .475) and TV viewing compared to computer use (*p* = .166).

Finally, we tested the moderating effects of the type of measurement of SB (self-report, *k* = 11 vs. objective measurement with accelerometry, *k* = 6). Only correlation coefficients for total SB time were used in this moderation analysis. This strategy was chosen to avoid confounding results with the effects of the type of SB. Results of the moderation analysis yielded a non-significant difference (*p* = .299) between estimates obtained for the two types of SB measurement.

Although four original studies employed a longitudinal design, we did not conduct a moderation analysis to test differences between cross-sectional vs. time-lagged effects. Such an analysis was impossible because only one longitudinal study provided time-lagged coefficients.

## Discussion

This study provides a preliminary synthesis and meta-analysis of evidence for associations between SB and anxiety symptoms. Results of the meta-analysis indicated that higher levels of SB were related to higher levels of anxiety symptoms, yet the estimate of the average effect was weak (weighted *r* = .093). The conclusions obtained from this meta-analysis are preliminary due to a relatively small number of studies included, their heterogeneity, and the inclusion of studies with cross-sectional designs. The systematic review indicated preliminary support for a significant association between SB and anxiety symptoms, with 51% of significant and positive associations (42.5% of non-significant associations, 6.5% of significant and negative associations). This moderate evidence, obtained in the synthesis of 31 studies is in line with findings of a previous review [31], presenting evidence obtained in 9 original studies.

A relatively small percentage (51%) of significant, positive associations between SB and anxiety symptoms was identified in the systematic review. This fact may be due to the methodology of the original studies. The majority of studies yielding non-significant associations were conducted with relatively small samples (with *N* < 100). Our meta-analysis shows that the average effect may also be small. Thus, the studies with small samples were probably underpowered to detect the associations between SB and anxiety symptoms. Future research targeting SB—anxiety associations should assume small effect sizes for a-priori power analyses.

The weakness of the overall association between SB and anxiety symptoms may have several causes. The association between SB and anxiety symptoms may be of indirect rather than direct nature, with a number of involved psychosocial and physiological mediating mechanisms. For example, in line with the displacement hypothesis [33, 34], it may be expected that a withdrawal form anxiety-reducing activities (such as physical activity, active social face-to-face interactions) is followed by SB. Therefore, SB that may constitute an avoidance behavior, exacerbating avoidance-related thoughts, that result in anxiety. Furthermore, a withdrawal from anxiety-reducing activities and subsequent SB engagement may result in lower self-esteem, which, in turn, may prompt anxiety symptoms (for the role of self-esteem see Smith et al. [35]). Future research should look more carefully into the underlying mediating mechanisms, instead of focusing solely on direct associations between SB and anxiety.

Another cause of weak associations between SB and anxiety may lie in the different operationalizations and different instruments used to assess SB and anxiety in the original studies. SB and anxiety were defined, operationalized, and measured in multiple ways, which poses a major challenge to comparability. Analyzed studies usually relied on a global index of SB, namely total SB time. Although this index is recommended, recent evidence suggests that physiological effects of SB may be better captured with other indicators, e.g. time composition (i.e., the relative proportion of total SB time, light-intensity physical activity, and moderate-to-vigorous-intensity physical activity [39]). Such indices would also allow for a more thorough validation of the displacement hypothesis, accounting for other energy expenditure behaviors. Moreover, due to the heterogeneity of operationalizations and measurement of anxiety symptoms we were unable to clarify to what degree the definitions and assessments of anxiety applied in original trials may have contributed to the overall heterogeneity of the estimates of the average effect.

Although the association between SB and anxiety symptoms was weak, its significance should be highlighted. Previous meta-analyses investigating the associations between SB and global indicators of mental health (i.e. emotional quality of life) showed a non-significant association [51]. Significant associations observed in this systematic review and meta-analysis suggest that SB may form links with specific aspects of mental health, such as anxiety symptoms. Further research should investigate if the strength of SB—mental health outcome depends on the type of the mental health outcome, that is anxiety, mood, somatoform, or sleep disorders.

We observed some differences between meta-analytic and systematic review findings. For example, the effect of age on the SB—anxiety symptoms relationship was supported by the systematic review, with significant associations emerging in 58% of associations tested among children and 48% of associations tested among adults. In turn, this meta-analysis indicated a trend for more consistent, significant associations among adults, compared to weaker associations among children/adolescents (*p* = .085). These discrepancies may be explained by our meta-analytic strategy which excluded studies with adults with a chronic mental or physical illness, as we identified no study with children/adolescents with a chronic illness. The moderating effects of age may further depend on the health status of the studied population. Future research should carefully investigate synergistic effects of age and health status.

The present study provides a preliminary synthesis of evidence which may inform clinical practice. Obtained findings, indicating that the observed effects are similar across groups differing in age and health, implicate the breadth of the target population for health promotion programs. Broad target populations may be a vector of successful implementation of health promotion programs [115].

A limitation of the present work refers to its inability to clarify the order in which SB and anxiety symptoms operate. Although theoretical models of stress resilience as well as displacement hypotheses [30, 33] suggest that SB precede and explain anxiety, it may be also assumed that anxiety may lead to a withdrawal from activities such as face-to-face social interactions, and thus allow for more time spent in SB. Our findings do not allow for any conclusions regarding the order of variables in this relationship, because the majority of included original studies had cross-sectional designs. Moreover, due to a limited number of studies focusing on children only or older adults only, we were unable to conduct a systematic investigation of age-related differences in the associations of SB and anxiety across the lifespan. The comparisons were made for broader age groups (children/adolescents vs. adults/ older people), therefore the conclusions referring to the effects of age should be considered as preliminary. Furthermore, analyses of the moderating role of age rely on a comparison of effects obtained in independent and heterogeneous cohorts. More research using longitudinal designs that would allow to establish the strengths of SB-anxiety associations across the life-span would provide more conclusive evidence for the existence of a moderating effect of age.

There are several other reasons for considering the present findings as preliminary. First, a small number of studies were entered into the meta-analysis. Second, the studied populations and indicators of SB and anxiety were of high heterogeneity. In particular, the results of the moderator analyses should be treated with caution as they were conducted with a small number of original studies which limits the likelihood of obtaining statistically significant findings. For example, the comparisons of subtypes of screen-related behaviors (computer use vs. playing with video/console games) were based on meta-analysis of 4 coefficients only, therefore any conclusions regarding the effect of the subtypes of screen-related behaviors are preliminary. Also, future studies should use more precise methods of assessing the content of screen time activities (e.g., using one’s mobile to play a game vs. social media use). Precise assessment would allow for a better identification of the subtypes of SB. The limited number of studies did not allow for a thorough test of combined moderating effects of age and health status. Other potential sociodemographic moderators, such as gender, were not analyzed because the original studies did not provide data allowing for the calculation of SB--anxiety association coefficients for men and women separately. The categorization regarding health status was suboptimal, as the two distinguished categories were very broad and comprised subcategories. In particular, the ‘chronic illness’ category referred to physical and mental health issues, whereas the ‘general population’ category included studies focusing on subsamples of general population (i.e., healthy pregnant women). Unfortunately, the number of studies was too small to conduct further moderator analyses (e.g. mental vs physical chronic illness). Consequently, the effects of health status should be further investigated before any generalizations are made. The use of self-reports to measure SB and anxiety symptoms may inflate the relationship between these two constructs. The value of the Egger test indicated a likelihood of publication bias, however, such values are typical for meta-analyses conducted with a limited number of studies [51, 116].

## Conclusions

Despite its limitations, this study provides a novel insight into the associations between SB and anxiety symptoms. The meta-analytic findings, based on 17 original studies enrolling children/adolescents and adults from the general population or with a chronic mental or physical illness, suggested that higher levels of SB are associated with higher levels of anxiety symptoms (the estimate of the overall average effect: *r* = .093). The associations remain largely similar, regardless of age, health status, SB operationalization and measurement. Trends for stronger SB—anxiety associations among adults (compared to children/adolescents) should be investigated further.

## Additional file

Additional file 1: Summary of studies included in the meta-analysis*.* Table displays r coefficients used in meta-analysis and coding for respective moderators. (DOCX 73 kb)

## Abbreviations

ASB: Accelerometry measured sedentary behaviors
CI: Chronic illness
CU: Computer use
GAD: Generalized anxiety disorder
MVPA: Moderate-to-vigorous physical activity
RT: Resistance training
SB: Sedentary behaviors
ST: Screen time
STAI: The Spielberger’s state-trait anxiety inventory
TV: Television
VG: Video games
