# Supplementary material for: Sedentary behaviors and anxiety among children, adolescents and adults: a systematic review and meta-analysis
Source: BMC Public Health. 2019 Apr 30;19:459. doi: 10.1186/s12889-019-6715-3 (PMC6492316; doi:10.1186/s12889-019-6715-3)
Supplement: Supplementary file 1 — Summary of studies included in the meta-analysis. Table displays r coefficients used in meta-analysis and coding for respective moderators. (DOCX 73 kb) [file 12889_2019_6715_MOESM1_ESM.docx]

Additional file 1. *Summary of studies included in the meta-analysis*

| **Study**  **(first author, year)** | ***r* coefficients used in meta-analysis^a^** | **Coding for^b^:** A – age group, B – health status, C – the type of measurement of SB, D – the type of SB, E – types of screen behaviours |
| --- | --- | --- |
| Asztalos et al., 2015 [54] | **Anxiety x Total sitting min/week: *r* = .02; *p* = .292** | 1. Adults 2. GP 3. Self-report 4. Total sitting 5. Excluded from the moderation analysis |
| Bampton et al., 2015 [59] | **Anxiety x Total weekly sedentary time: *r* = .19; *p* < .001** | 1. Adults 2. GP 3. Self-report 4. Total sitting 5. Excluded from the moderation analysis |
| Feng et al., 2014 [71] | **Anxiety x ST: *r* = .04; *p* = .195** | 1. Children/adolescents 2. GP 3. Self-report 4. ST 5. Excluded from the moderation analysis |
| Gibson et al., 2017 [75] | **Combined index of: *r* = .04; *p* = .81:**   1. **Anxiety x Sitting hours weekday: *r* = .14; *p* = .256** 2. **Anxiety x Sitting hours weekends: *r* = -.06; *p* = 636** | 1. Adults 2. GP 3. Objective 4. Total sitting 5. Excluded from the moderation analysis |
| Gunnel et al., 2016 [76] | **Anxiety x Sedentary minutes: *r* = .17; *p* = .009** | 1. Children/adolescents 2. GP 3. Self-report 4. ST 5. Excluded from the moderation analysis |
| Janney et al., 2013 [80] | **Anxiety x Sedentary minutes: *r* = .22; *p* = .142** | 1. Excluded from the moderation analysis 2. CI 3. Objective 4. Excluded from the moderation analysis 5. Excluded from the moderation analysis |
| Kroeders et al., 2013 [84] | **Anxiety x % lying-sitting: *r* = .13; *p* = .601** | 1. Excluded from the moderation analysis 2. CI 3. Objective 4. Excluded from the moderation analysis 5. Excluded from the moderation analysis |

| Liu et al., 2016 [86] | **Combined index of: *r* = .01; *p* = .183:**   1. Anxiety x ST (TV): *r* = -.001; *p* = .908 2. Anxiety x ST (electronic games playing): *r* = .02; *p* = .005 | 1. Children/adolescents 2. GP 3. Self-report 4. ST 5. TV and electronic games playing |
| --- | --- | --- |
| Maras et al., 2015 [89] | **Anxiety x ST: *r* = .05; *p* = .007**  Combined index of (*p* = .213):  1) Anxiety x computer games plying: *r* = .06;  2) Anxiety x video games playing: *r* = -.01 | 1. Children/adolescents 2. GP 3. Self-report 4. ST 5. Electronic games playing |
| Opdenacker, & Boen, 2008 [91] | **Anxiety x Total sitting min/week: *r* = .46; *p* < .001** | 1. Adults 2. GP 3. Self-report 4. Total sitting 5. Excluded from the moderation analysis |
| Padmapriya et al., 2016 [92] | **Combined index of: *r* = .08; *p* = .01:**   1. Anxiety (STAI State subscale) x Total sitting time: *r* = .09; *p* = .01; 2. Anxiety (STAI Trait subscale) x Total sitting: *r* = .08; *p* = .01; 3. Anxiety (STAI State subscale) x TV: *r* = .12; *p* < .001; 4. Anxiety (STAI Trait subscale) x TV: *r* = .07; *p* = .03 | 1. Adults 2. Excluded from the moderation analysis 3. Self-report 4. Total sitting 5. TV (combined indexes no. 3 and 4: *r* = .10; *p* < .01 ) |
| Park et al., 2017 [93] | **Anxiety x % of SB: *r* = .39; *p* < .01** | 1. Adults 2. CI 3. Objective 4. Total sitting 5. Excluded from the moderation analysis |
| Rebar et al., 2014 [94] | **Anxiety x Overall sitting time: *r* = .15; *p* < .001**  Anxiety x computer using: r = .10; *p* < .001  Anxiety x TV: r = .07; *p* = .003 | 1. Adults 2. GP 3. Self-report 4. Total sitting 5. TV and computer using |
| Straker et al., 2013 [98] | **Anxiety x ST: *r* = .02; *p* = .597** | 1. Children/adolescents 2. GP 3. Self-report 4. ST 5. Excluded from the moderation analysis |
| Teychenne & Hinkley, 2016 [100] | **Anxiety x ST: *r* = .11; *p* = .007**  Anxiety x computer using: r = .14; *p* = .001  Anxiety x TV: r = .07; *p* = .119 | 1. Adults 2. GP 3. Self-report 4. ST 5. TV and computer using |
| Vallance et al., 2015 [104] | **Anxiety x Weekly total sedentary time: *r* = -.09; *p* = .222** | 1. Excluded from the moderation analysis 2. CI 3. Objective 4. Excluded from the moderation analysis 5. Excluded from the moderation analysis |
| van Roekel et al., 2016 [105] | **Anxiety x Total sedentary time: *r* = .03; *p* = .774** | 1. Excluded from the moderation analysis 2. CI 3. Objective 4. Excluded from the moderation analysis 5. Excluded from the moderation analysis |

*Note.* ST – screen time; GP – general population; CI – chronic illness; STAI – the Spielberger’s state-trait anxiety inventory.

^a^ Coefficients presented with bold were used to obtain total average effect and effects of moderation analyses (for moderators from A-D).

^b^ Coefficients for moderator analyses for E – the type of ST are presented in column ‘*r* coefficients’ without bold.

^*^*p* < .05; ^**^*p* < .01; ^***^*p* < .001; n.s. - not significant.
